# Cognitive enhancement kept within contexts: neuroethics and informed public policy

**DOI:** 10.3389/fnsys.2014.00228

**Published:** 2014-12-05

**Authors:** John R. Shook, Lucia Galvagni, James Giordano

**Affiliations:** ^1^Philosophy Department and Graduate School of Education, University at BuffaloBuffalo, NY, USA; ^2^Bruno Kessler FoundationTrento, Italy; ^3^Neuroethics Studies Program, Pellegrino Center for Clinical Bioethics and Department of Neurology, Georgetown University Medical CenterWashington, DC, USA; ^4^Human Science Center, Ludwig-Maximilians UniversitätMunich, Germany

**Keywords:** ethics, neuroethics, cognitive science, neuroscience, cognitive enhancement, culture, public policy

## Abstract

Neurothics has far greater responsibilities than merely noting potential human enhancements arriving from novel brain-centered biotechnologies and tracking their implications for ethics and civic life. Neuroethics must utilize the best cognitive and neuroscientific knowledge to shape incisive discussions about what could possibly count as enhancement in the first place, and what should count as genuinely “cognitive” enhancement. Where cognitive processing and the mental life is concerned, the lived context of psychological performance is paramount. Starting with an enhancement to the mental abilities of an individual, only performances on real-world exercises can determine what has actually been cognitively improved. And what can concretely counts as some specific sort of cognitive improvement is largely determined by the classificatory frameworks of cultures, not brain scans or laboratory experiments. Additionally, where the public must ultimately evaluate and judge the worthiness of individual performance enhancements, we mustn’t presume that public approval towards enhancers will somehow automatically arrive without due regard to civic ideals such as the common good or social justice. In the absence of any nuanced appreciation for the control which performance contexts and public contexts exert over what “cognitive” enhancements could actually be, enthusiastic promoters of cognitive enhancement can all too easily depict safe and effective brain modifications as surely good for us and for society. These enthusiasts are not unaware of oft-heard observations about serious hurdles for reliable enhancement from neurophysiological modifications. Yet those observations are far more common than penetrating investigations into the implications to those hurdles for a sound public understanding of cognitive enhancement, and a wise policy review over cognitive enhancement. We offer some crucial recommendations for undertaking such investigations, so that cognitive enhancers that truly deserve public approval can be better identified.

In its disciplinary stance and practice, neuroethics acknowledges the most sufficiently confirmed theories of the neural and cognitive sciences as (provisionally) accurate in an overriding manner. Indeed, neuroethics should not ignore or set aside such theories if/when these prove to be inconvenient for, or incompatible with, practical applications, principled values, private intuitions, or popular common sense. Nor should these theories be muted when neuroethical engagement of real-world issues, questions and problems are needed. The actual functions of the brain, as best as can be described at present, is—and must remain—fundamental to any and all neuroethical discourse and deliberations.

In light of this, we argue that neuroethical inquiries into cognitive enhancement should establish the crucial role for context, especially socio-cultural contexts to conceptions of the “cognitive”, so as to better define the realities of neuroethical debates about cognitive performance enhancement (and enhancers). We write in support of investigations into putative neuro-enhancers which adopt the theoretical stance that hoped-for improvements cannot be evaluated in isolation from human activities that provide the meaningful context to any alteration in performance. At key points of our argument we appeal to empirical studies examining performance alterations, to exemplify our concern for due attentiveness to real-world situational conduct as possible enhancers are tested and utilized. Where relevant contexts to neuroscientific inquiry and information utilized for normative purposes are taken seriously, the work of formulating and guiding neuroethical quandaries would be appreciably strengthened.

With our argument for this role of context established, we then outline crucial policy considerations regarding neuro-cognitive enhancement, and raise some warnings about over-eager advocacy for enhancement. While remaining cautiously optimistic toward opportunities for cognitive enhancement, we insist upon fostering public understanding of the issues, and upon development of sound public policy. In this way we join the ranks of other neuroethicists who have voiced similar views and concerns (Illes and Bird, 2006; Levy, 2011; Farah, 2012; Gunson, 2012; Fitz et al., 2014; Maslen et al., 2014b; Racine et al., 2014).

As a discipline and set of practices, neuroethics must utilize the best cognitive and neuroscientific knowledge to shape incisive discussions about what could possibly count as enhancement in the first place, and what should count as genuinely “cognitive” enhancement. Where cognitive processing and mental life is concerned, the lived context of psychological performance is paramount. In the absence of any nuanced appreciation for the control which performance contexts and public contexts exert over what “cognitive” enhancement could actually be, enthusiastic promoters of cognitive enhancement can all too easily depict brain modifications as being good for individuals and for society. Such claims are not unaware of serious hurdles—hurdles noted in oft-heard observations and express concerns—that may impede enhancement derived from neurophysiological modifications. Yet those observations are far more common than penetrating investigations into the implications that such hurdles may evoke—both for the sound public understanding of cognitive enhancement, and for wisely informing policy to guide and govern cognitive enhancement. To wit, we offer what we maintain to be crucial recommendations for undertaking such investigations, so as to better identify cognitive enhancers that truly deserve public approval and policy support.

Civics and general ethics can be idealistic, but neuroethics should not be unrealistic, and must be liberated from ethical theorizing done in ignorance of the most contemporary understanding of the structure and function of the brain. In this way, neuroethics must strive to comprehend the genuine basis of conceptions of self, society, and morality, and rely on changes or replacements to those conceptions when and where scientifically warranted (Shook and Giordano, 2014).

## Enhancement standards

Bioethicist Thomas Murray identifies two primary meanings to the term “enhancement”: first, “to advance, augment, elevate, heighten, increase”; and second, “to increase the worth or value of.” (Murray, 2007) In both definitions, context is axiomatic. Numerous scholars have similarly noted the “metric” and “normative” dimensions of this term. For an organism such as a human being, enhancement implies opportunities to improve capacities or abilities—features that can be simultaneously measurable and valuable, and possibly moral, as well. Structure and function cooperate and even interfuse, even as they have distinct implications for ethicality, and this can confuse discussions of enhancement, in general, and of “cognitive enhancement” more specifically.

Hence, it is important to ask whether a particular modification is responsible for altered performance of a specified task. If it is, then that physiological modification is a *performance modifier*, and if that change is regarded as positive relative to some normative standard, then we can refer to it as a *performance improver*. Furthermore, if we call a particular activity an “intellectual” task, then we are really talking about an *intellectual enhancement* for performing that task. This physiological modification may be labeled as an “intellectual enhancement” in an easy, colloquial manner of speaking, although at this point in the description, a scientific understanding of the brain or intellectual capacities is not yet involved.

In light of that science, however, it can no longer be enough to simply track cognitive functions and the resulting performance on particular tasks to verify enhancers. An alteration to a physiological process associated with cognition can be measured and compared against some organic standard. Has enhancement occurred? It is still too soon to say; a pre-set physiological standard is never simply a “given” normative standard. Detecting a physiological alteration within an individual requires an individual-relative standard against which to measure that alteration, to be sure. But what may be expected from one person needn’t expected from others. Setting someone’s neurological functioning as the standard for any human brain’s “normal” functioning, so that a baseline for cognitive functioning can be established in preparation for detecting cognitive improvement done to anyone, is quite another normative decision. One that normality standard is put in place, then actual cognitive function (for the processing and integration of various types of sensations, memories, emotions, subconscious valuations, and so on) can be estimated and compared against some standard of normality. Even after this has been done, it still may be premature to say whether or not the evoked changes represent an enhancement; reliable cognitive performance (for one’s overall management of life activities and achievements) must be judged in light of some *ethical* standard. Just as human-normal performance can’t be reduced to any single individual’s functioning, ethically responsible enhancement cannot be reduced to any superiority over what has been conventionally set as generically normal for “average” humans.

Standards at the three levels of individual physiology, human-generic normality, and sensible ethicality all compete for prominence where definitions of “enhancement” are concerned. Furthermore, it doesn’t help that the complexities of the nervous system can permit odd scenarios in which an increase in physiological function(s) might diminish cognitive ability; and diminishing a specific type of cognitive function might be conducive to optimizing a person’s actions or general well-being (Earp et al., 2014). Rigidly demanding only one standard, or one direction by that standard, with which to dictate enhancement is a stubborn path to take, and one that we assert any rational approach to neuroethical analysis and discourse should avoid.

Letting the concept or term “enhancement” stand for any non-therapeutic benefits conferred by an intervention is a common way to avoid taking any (if not all three) standards seriously. Does enhancement begin when a medical treatment exceeds the usual dosage or typical extent of repair? Perhaps enhancement refers to those instances where treatment yields physiological functioning beyond the normal range. Or, enhancement might entail evoking superior performance that lends distinct advantages to life. Arguing over these options overlooks the mistake that enhancement can begin where therapy ends. It is a mistake that is easy to make.

Therapeutic medicine simplifies its standards because it takes all of humanity to be its proper field of work; a good treatment for a health deficiency generically helps any patient suffering from that problem. So long as the reference class remains “humanity,” then a physiological “average” functioning can be equated with generic normality, and there would only be “disease treatments” (aiming towards normality) and “enhancement treatments” (aiming beyond normality). However, patients aren’t so generic in the real world. Broad culture and local society are contexts that always exert influence.

For example, what can count as normality and abnormality within a particular culture might not obtain for all of humanity too. But, if that culture’s influence (viz.—power) is sufficiently strong, clinicians, patients, publics and governing bodies might not necessarily notice, or care (or feel empowered to act even if they did). Furthermore, any social group within that culture could come to regard itself as the proper reference class, expressly if that group enjoys some status and/or privilege. When that social group requests medical treatment, it is set in terms of what counts as “group normal” rather than just “culturally normal” or “normal for humanity.” For example, when middle-aged privileged men take their reference class as “adult men like us”, they surely aren’t thinking about “all human males on the planet between the ages of 18 and 80.” Nor are they taking their reference class to be people very much like them, such as “successful men between 45 and 65.” Instead, what counts as “normality” is the reference class that these men desire to be, perhaps something like “healthy guys in their 30s-50s.” So, in effect they want what counts as “subgroup optimal.” Growing approval among a subgroup about using a drug or device off-label lends credence towards that intervention’s status as an “enhancer”, even if that subgroup expects more than just intellectual performance (Hildt et al., 2014; Wade et al., 2014). Interventions can transition from enhancers back to treatments, as well. If a culture’s medicine proves willing, then treatment for achieving subgroup optimality could be labeled as medical “therapy”, rather than enhancement.

Neuroethics must take close notice of: (1) the kinds of standards applied for determining enhancement; (2) the chosen reference class serving as the background against which enhancement would stand out; and (3) the selection of “normality” or “optimality” as the envisioned goal to enhancement. Medicine’s traditional focus on generic remedies for universal application to all humanity is not the best (or perhaps even a viable) framework for identifying and classifying enhancements. Cultural inheritance, group socialization, personal values, and physiological factors are each and all necessarily involved when defining and addressing enhancement. The advent of “personalized” medicine aiming towards the individualization of diagnostics and treatments should raise awareness across neuroethics that specifics will matter to ever greater degrees in the future.

## Enhancing cognition in context

The temptation to regard cognition as an entirely neurophysiological matter, amenable to objective study, definition, and measurement, isn’t just a symptom of overreaching reductionism or scientism. Frustration with too much context can set in for anyone reconciled to cognition’s reliance on brain functioning. If cognition is, in some sense, objectively present as subjects undergo experimental study, then it could be objectively modified. Researchers would be able to determine when and how cognition is improved when compared against some pre-set standard of cognitive ability. Serious attention to cognitive enhancement came to the fore as a consequence of experimental facilitation of cognitive ability, with due caution leveraged against exaggerated claims of capability, meaning and utility. (Metzinger and Hildt, 2011; Sahakian and Morein-Zamir, 2011; Sandberg, 2011; Chatterjee, 2013; Cohen Kadosh, 2013; Hildt and Franke, 2013). Hard lessons learned from pharmaceutical studies apply to any sort of performance effects produced by alteration of brain structure and function (Luber, 2014).

Neuroethical attention must be paid to wider contexts of neurological manipulation, beyond the fairly objective and narrow ways that cognitive performances can be adjusted in desired directions. Determining if a neurological intervention can actually produce a desired enhancement is one thing; ascertaining that some sort of adjustment is truly cognitive (in the expected manner) is quite another, and these distinctions deserve priority. Imitating medicine’s quest for therapies that have universal utility for anyone suffering from a generic health problem is no longer a wise undertaking for the application of 21st century biomedical advancements. As well, we maintain that it is equally unwise to promote enhancements as if they could be universally beneficial for generic cognitive improvements to anyone’s intellectual performances. Indeed, we argue that there may not be such a thing as a “generic enhancement to cognitive performance.” A major reason for this involves cultural contexts. Two people from two different cultures, or even two people from two subgroups of the same culture, may not necessarily agree on what is cognitively adjusted by some alteration of neurological function. Thus, neuroethical inquiry cannot avoid an interpretative circle: some group of people ascribe a “function” to a cognitive process in service of a task that is considered to be “normal”—but this is a social imposition of normality on a neurophysiological process. In this way, performance, not neurophysiology in isolation, decides functionality, and what counts as “normal”.

For illustration, consider an analogy: suppose a practical way to increase muscle mass (without deleterious side effects) is offered as a general “athletic enhancer” that could be used by anyone. Athleticism depends on one’s musculature, surely, so given this rationalization, more muscle should enable more athleticism. But muscle mass alone does not equate with athletic ability (or in some cases even potential ability!) For example, one can take anabolic-androgenic steroids (AAS) to augment muscle mass. As matter of fact, these very likely will lend something of an “edge” to (important) dispositions and characteristics necessary for improved athletic performance (i.e.,—muscle size and strength; Llewellyn, 2010). However, the underlying premise is that the agent is increasing specific qualities of muscle (e.g.,—diameter of muscle fibers, contractile force, etc.) that have been shown to be operative in a number of athletic events.

Herein though, are important caveats. While an AAS may yield mass and strength gains, these are only preparatory for “training effects”, because an athlete must still train for a particular sport. AAS can facilitate that training, but if training is conducted improperly, less success at a sport is a likely result. Furthermore, different physiological agents can elicit distinct effects. Some will enable gains in muscle mass but not necessarily facilitate definition; others will be more lipolytic, and produce lean, muscular density, but will not greatly increase mass, and so forth. Also, AAS does little for aerobic endurance *per se*, just as an endurance-facilitating agent (such as erythropoietin, EPO) does little for mass or strength. (consult Llewellyn, 2010). The adage is: The right agent for the right effect.

These points account for the ample evidence—and practical wisdom—indicating that if one wants to become proficient in a particular sport, then it is necessary to vigorously train in that sport. There are generic athletic training exercises, but each sport must evaluate their utility. For example, cross training can lend overall benefits to components of athleticism, but it doesn’t necessarily permit direct performance gains peculiar to each sport. Only after specific kinds of athletic performances, and the individual athletes performing them, are identified and targeted, would an intervention be intelligently developed and employed to exert positive effect(s) within selected contexts. Expanding upon this example of sports performance, we may expect that most types of neurological interventions intended for the enhancement of performances displaying much complexity may only work best in conjunction with cognitive training regimens. Not only must any trials confirming a modification for cognitive improvement involve successful routine practice under controlled conditions, only implementing that modification in conjunction with strenuous performance training result in the practical enhancements to performances actually valued outside of any laboratory.

More generally, it is naïve to suppose that a compensatory adjustment, much less an enhancing adjustment, could be generically assigned validity across all of humanity. Even best-case scenarios must remain stubbornly diffuse. Calling a performance test a “cognitive performance test” and observing that individuals who are subjected to intervention “x” perform better doesn’t mean that some purely cognitive functioning has been isolated and targeted as the improved factor. Fortunately, careful research is hardly so naïve, as recent exemplars have noted (Pringle et al., 2013). The lesson is that no one pondering cognitive enhancement should assume that higher cognition can occur in some “pure” forms, no matter how specific the task. To begin with, multiple affective and motor processes are interfused with the functional components that are operative in executive control. In turn, executive control is interfused with every sophisticated practice acquired during childhood and adolescence. This is especially the case when dealing with higher cognition manifesting in social and moral behaviors (Shook, 2012; Specker et al., 2014).

Enculturalization takes advantage of advanced executive control for instilling specialized task performances, such as learning mathematics and logic. It is no paradox that the more cognitively abstract the task, the more it has a cultural rather than a purely biological basis; hence such tasks are very much subject to the vagaries of cultural history and practice. Something as simple as conceptualizing number and amount has been shown to be culturally variant (Núñez, 2011). Similarly, memory performance has been shown to be culture-dependent and -influenced (Gutchess et al., 2011; Hewer and Roberts, 2012). Cultures contribute to cognition as much as cognition contributes to culture (Han and Pöppel, 2011; Ishii, 2013; Kim and Sasaki, 2014). Even context is contextual as far as cognition is concerned, since the developing sensitivity towards, and responsiveness to, environing interpersonal context displays cultural variability (Imada et al., 2013).

The contextual factors raised here are not posed to endorse a thorough relativism or dismissive eliminativism about potential enhancers. Cognitive enhancement can be quite real, when and where it is created. To be sure, confirmable cognitive enhancements can be achieved because improved cognitive (i.e.,—intellectual and/or emotional) performances by selected and trained participants can be measured under controlled conditions. Generally speaking, under sufficiently similar conditions, similarly altered people having enough in common will perform similarly, all other things being equal. What more could be expected from science?

## Enhancement in public contexts

Desires to “improve the human condition” conjure proposals for a proverbial “rising tide” of neuroscientific and neurotechnological modifications that will “raise all boats”—and brains. But when realistically looking ahead, unavoidable questions loom: How much can humans be enhanced without deforming or destroying aspects of the social or natural world on which life relies? and, Will human character and moral progress be sustained if hopes for enhancement become realized? Some have supported a duty to urge enhancers and even intervene with required enhancement, once we can apply a safe and effective intervention. However, our comprehension of long-term consequences is limited, and encouraging (what may be long-lasting) modifications without ensuring equally durable individual welfare is reckless (Rossi et al., 2013).

Shall the position of the responsible individual prevail instead? Letting individuals choose for themselves is no less reckless. Even when individual benefits can be guaranteed, it must be asked: who should receive them? The answer, “All who can benefit,” is no answer at all, because it will not be the case that everyone will have the same, or even similar, access at the same time. Differential access is inevitable in a world of finite time and resources. Such differential access is *prima facie* unjust, as those who already possess certain traits, attributes, and/or resources will likely and quickly get even more. Hence, essential concerns for distributive justice arise from the position of society at large. The distribution of improved health and lifestyle status, and even improved moral status, will always be a social concern (Buchanan, 2011; Douglas, 2013).

Worries over distribution cannot—nor should not—be easily dispelled. Those with the least assets are those most unlikely to get access to state-of-the-art scientific and technological interventions. It is unrealistic to assume that some massive shift in the social architectonics of medical resource allocation will occur so as to allow neuroscience and neurotechnology to close the gap between those who “have” and those who “have not.” Given this reality, does everyone really want a society where the people getting the most enhancement(s) are precisely those enjoying great wealth? The prospect of cognitive enhancement surely highlights this worry: intelligence does what character directs, and the kinds of characters getting so wealthy in a society may not be the people to be trusted with even more intelligence—and power. Proponents of unlimited access to enhancement simply point the way toward an unbalanced distributive scheme. Contests between rival distribution methods can be debated in ethics, but they get adjudicated in politics.

Hence, entering the realm of politics becomes unavoidable. The politics surrounding access to enhancement will be intense. Of equal importance is the temptation to use brain science within agendas of political power to control fundamentally biological aspects of individuals’ and communities’ existence (invoking what Foucault referred to as biopolitics; see Foucault, 2008; Anderson et al., 2012). Bioethical and neuroethical analyses cannot avoid addressing science as a public good; ethics as a search for the good and the right; and politics as the participation of citizens in decisions about the guidance of public order. As public debate over the impact(s) of enhancement interventions accelerates, the search for principled guidelines has ensued, and neuroethics has become ever more involved (Bostrom and Sandberg, 2009).

Irrespective of whether enhancement is regarded as a bountiful cornucopia or a ticking bomb, the differing contexts of enhancement radically transform its biopolitical status. Recall from a previous section our attention to the choice among physiology, normality, and ethical standards for identifying what counts as enhancement. Experimental medical research focusing upon physiological alterations (typically) emphasizes interventions for those who are the most unhealthy. Policy tends to approve funding for basic research if and when it could soon help those with the most severe, and/or epidemiologically extensive, health conditions. These prioritizations wouldn’t work in the realm of enhancement for three reasons. First, a traditional approach to funding and engaging research would tend to leave most enhancements on the theoretical drawing board. Second, while there may be desires for expensive advanced research into fundamental neurological mechanisms that can be targeted for cognitive performance enhancement, unless these approaches can be ascribed to incur some “therapeutic” benefit against an identified disease, disorder or (medical) condition, financial and administrative support for broad scale research and translation of outcomes and products would tend to be lacking. Third, while there may be a viable—and perhaps growing—market for certain cognitive performance enhancements, it is difficult to generate the funding necessary to support and sustain exploratory research required for translation to safe commercial technologies (unless developed and marketed as “non-medical” products such as toys and games, which then raises the specter of inapt and/or unsound development, distribution and use; see, for example, Giordano and DuRousseau, 2011; and Plischke et al., 2011).

A related issue is the contemporary medical endorsement of interventions that restore or sustain “normality.” Explicitly and implicitly this position conforms to socio-cultural requirements that all people should seek and exhibit “normal” functioning, rather than (what is regarded to be) abnormal or anti-social conduct that deviates from socially established standards. What posture should be assumed when (a) certain people seek optimal functioning in pursuit of what they personally deem as the apex of the good life, and/or (b) society sets requirements that individuals in special roles (such as physicians, pilots, peace officers, or warfighters) must attain some level of optimal functioning? (Giordano et al., 2013; Goold and Maslen, 2014) Medicine’s laudable work in service of living a good life isn’t automatically extendable to living a great life, or to achieving great performance in a socially-sanctioned service. Justifications for specialized enhancements for enabling idiosyncratic lifestyles or for extraordinary public service will not necessarily be obtained in and from medical principles.

A second set of examples arise from our earlier discussion of the cultural relativism inherent to the precise identification of cognitive improvements. Medicine’s due caution with clinical application, watching carefully for deleterious health and lifestyle side-effects, relies upon cultural consensus about what constitutes “normal” performance in daily life (Gini et al., 2010). Those seeking significant enhancements, by contrast, won’t be interested in conforming to cultural norms about ordinary performance, and medicine may not be able to restrain them. When the recipient of an enhancement is achieving extraordinary performance levels and feeling empowered to transgress cultural expectations in the name of greatness (despite the risks), what social institution or cultural tradition can and will reign-in their pursuits?

Evidently, society turns to law for such proscriptions. Yet, here it becomes necessary to ask how restrictions of, and prohibitions against certain types and extents of enhancement will be determined. Targeting concrete neurological modifications for legal bans (i.e.,—imitating medical bans of performance enhancing substances for professional athletes, and/or scheduling certain drugs) has the merit of objective verification. But this only spurs those seeking improved types of cognitive performance to find alternative physiological methods not yet banned or detectable, and the chase is begun anew.

## Policy priorities and the role of neuroethics

Frustration over excessive contextualization is a perennial complaint. Simplifying matters can seem attractive when modest advances require prompt address and short-term priorities are within reach. Simplification would be possible if the construct and term “enhancement” satisfied defined and pragmatic scientific and ethical criteria. That way, any continued debate would be centered on those improvements that were already deemed to be fairly good for people in general, so far as could be scientifically and ethically determined. Warnings are certainly in order that current enhancement interventions rarely prove to be wholly effective or without deleterious effects. Unsurprisingly, wide agreement among scientific, ethics, and policy communities can be found on the view that enhancing interventions shouldn’t be counter-productive or harmful to overall health.

However, we posit that practical risk-benefit analyses aren’t entirely sufficient. Detailed ethical scrutiny is required before any such practical improvements can be classified as good enhancers. It is wise to demand that putatively enhancing interventions do not diminish self-control or autonomy, degrade personal growth or self-worth, or diminish life-management and social skills (de Melo-Martín, 2010; Allhoff et al., 2011). These demands can be reasonably placed upon envisioned enhancements, even if they aren’t applied so stringently to proven medical therapies. Improvements towards health are usually consistent with personal empowerment, and the consequences of restoring normal functioning are largely understood. By contrast, the longer term effects of experimental enhancements, especially cognitive performance enhancements, on the psychological self and internal self-conceptions and motivations are among the least predictable and understood aspects of this issue. Ethics is rightly concerned about the vital capacities for autonomy, dignity, and morality. All the same, as we have noted, setting high standards for cognitive performance enhancing interventions need not cast dark suspicions upon the persistent search for, and studies of enhancements. A number of scholars have advocated for practical and ethical standards while endorsing the pursuit of enhancement (including Buchanan, 2011; Glannon, 2011; Giordano, 2012; Heinrichs, 2012; Clark, 2014; Maslen et al., 2014a). In short, the goal is to develop helpful interventions able to meet these high standards.

If such normative thresholds are maintained, public and regulatory approval could be a helpfully expedited matter. But approval may not be automatic. Labeling an intervention as an “enhancement” once it makes individual lives demonstrably better can’t be the final hurdle before regulatory approval (Giordano and DuRousseau, 2011). One further—and arguably major factor—that cannot be omitted is the wider public context. We believe that this is where the broadest and deepest deliberations over the wisdom of enhancement should occur. We are forced to ponder what shall be done when sound public priorities cannot automatically approve genuinely ethical enhancements.

Policy principles should be well informed, ethical, and just. When some reliable enhancements are deemed safe and effective, and seem capable of promoting the good life, why wouldn’t they be approved through policy and law? Here, it is important to appreciate that sincere advocacy of genuine individual enhancers could still be under-informed, potentially unethical, and possibly unjust. In those cases, public wisdom should lean against approval.

In this regard, two issues must remain distinct: First, it must be asked, and determined, if an intervention is a genuine enhancer. Second, if it is, then it must be asked if this enhancer is something that sound policy can approve and sanction. The criteria by which an enhancement is deemed conducive for the “good life” cannot be the same criteria that are used to determine whether to approve it. In the open space of public deliberation, it must be possible that sound policy can proscribe or prevent something that is presently understood to be reliably conducive to the “good life”.

Here we avoid assumptions that knowing what is conducive to the good life for each person constitutes knowing what is ethical and wise. We also avoid the position that knowledge about what is conducive to the “good life” for everyone constitutes knowing what is ethical and wise. Rather, we posit an alternative stance. We argue that (1) well-informed policy would use more information than just the scientific facts about a performance enhancer promoting the “good life”; (2) ethical policy would use other ethical criteria beside simple promotion of the “good life” (individually or collectively); and (3) just policy may prefer a stable and well-ordered society that isn’t advancing the individual or collective “good life” quite as fast as could be technologically possible (or imagined by technophiles).

Gazing upon the stance we propose, eager advocates of enhancement might ask why objective scientific facts couldn’t or shouldn’t lead the way, especially when cognitive performance enhancement seems so modest, practical, and generically useful? In doing so, they appear to endorse the general ethical guideline that:

A sound policy decision will always approve what, in light of ascertainable scientific facts, can be expected to be an enhancement to an individual that is conducive to what “our society” regards as the “good life.”

This guideline does not represent our position; we deem it unwise and replete with confusions, and when it appears to be doing the real work behind hasty encouragements of cognitive performance enhancement, we deplore it. Whether this is the actual view of any bioethicist or neuroethicist, or just a caricature for academic target practice, we cannot really say, because few scholars have explicated their meta-ethical presumptions. We do assert, however, that this stance is inadequate to meet the urgent complexities and contextualities inherent to authentic human life as we all must actually live it. In fact, there is little that is genuinely neuroethical in it. Our call for a neuroethics that takes context seriously, especially where cognitive performance enhancement is the issue, isn’t merely fodder for academic debate.

Sarewitz and Karas (2012) outline several different approaches that can be adopted in order to make choices and decisions about cognitive enhancing technologies. Among those, our view aligns with the “optimistic” approach, via engagement of a managed technological optimism that best represents our position as relevant to ethical decision-making processes and public policies in this field. We endorse continued research into cognitive performance enhancements. We also call for the need to optimize definitions of any/all concepts and terms, and to equally define the contexts in which any cognitive task optimization can/would occur. Only from that point can one be optimistic that a progressive, non-static concept of the human and human function will be realistically entertained and enhanced, both practically and ethically. This position takes a pluralistic, democratic approach towards options of emergent (rather than merely proscriptive) governance, and the final section of this essay points to ways that neuroethics can play a supportive role.

We posit that a contextualized neuroethical outlook allows for better-informed approaches, utilizing all relevant interdisciplinary input, for considering what therapies and enhancements could be. It permits neuroethical deliberation to rise above local conventionality and a single social ethos, to instead survey the rich cultural diversity of human self-understandings and dynamic cognitive capacities. And, it encourages neuroethics to render verdicts against destabilizing and unjust procedures in policy debates that rashly extend medical models beyond their proper functioning.

This neuroethics isn’t proscriptive, nor does it seek to uniformly obstruct enhancement. In its naturalistic basis, it establishes grounds to view the human as engaging biology (through intellectual and physical tools) to optimize survival and flourishing in changing ecologies. And in its appreciation for the human as a bio-psychosocial organism it engenders an interdisciplinary approach (conjoining anthropology, sociology, economics, and political science) to depict and address ethical issues within the contexts in which human activities are conducted. Thus, in the spirit of cognitive enhancement itself, neuroethics as a discipline—and in its methods, approaches, and practices—should embody and enable greater human self-understanding, and improve our public deliberations over the many dimensions of life that we treasure.

## Conflict of interest statement

The authors declare that this research was conducted in the absence of any commercial or financial relationships that could be construed as a potential conflict of interest.
